# Developing Iranian primary health care quality framework: a national study

**DOI:** 10.1186/s12889-019-7237-8

**Published:** 2019-07-09

**Authors:** Ramin Rezapour, Jafar Sadegh Tabrizi, Mostafa Farahbakhsh, Mohammad Saadati, Hossein Mashhadi Abdolahi

**Affiliations:** 1 Iranian Center of Excellence in Health Management, School of Health Management and Medical Informatics, Tabriz University of Medical Sciences, Tabriz, Iran; 2 Tabriz Health Services Management Research Center, Health Management and Safety Promotion Research Institute, Tabriz University of Medical Sciences, Tabriz, Iran; 3 Research Center of Psychiatry and Behavioral Sciences, Tabriz University of Medical Sciences, Tabriz, Iran; 4 Road Traffic Injury Research Center, Tabriz University of Medical Sciences, Tabriz, Iran

**Keywords:** Quality assessment framework, Quality of care, Quality indicators, Primary health care

## Abstract

**Background:**

Providing comprehensive and high-quality services is one of the most important goals of the health systems and a basic principle for Universal Health Coverage (UHC). Fulfilling this important task would be feasible through continuous evaluation and improvement of the health services quality. The aim of this study was to develop a framework for quality assessment of Primary Health Care (PHC) in Iran’s health system.

**Methods:**

This study is a literature review which continued by a qualitative research. The extracted quality dimensions and indicators for initial screening were reviewed and discussed in two panel meetings attended by the experts with regard to the current package of health system in Iran. Using Delphi method, the dimensions and Quality Indicators(QIs) were evaluated and approved by 39 national health professionals in two rounds. Finally, after 4 panel sessions at ministerial level, the selected QIs were categorized in form of the final dimensions of the quality of care.

**Results:**

The literature review emerged 13 Primary Health Care Quality Assessment Frameworks (PHCQAF) including 20 and 698 QIs. Delphi study resulted in developing Iranian PHCQAF comprising 7 dimensions and 40 QIs. Among these, 8 QIs of the dimension of access and equity, 5 QIs of safety dimension, 2 QIs of efficiency dimension, 13 QIs of effectiveness dimension, 2 QIs of patient-centeredness dimension, 3 QIs of governance dimension and 7 QIs of appropriateness dimension were presented.

**Conclusions:**

The presented PHCQAF can be used as a comprehensive and practical tool for continuous improvement of the quality of PHC services at local, national and regional levels. Moreover, it can give some useful information to the health managers and policy makers on how the services are provided.

**Electronic supplementary material:**

The online version of this article (10.1186/s12889-019-7237-8) contains supplementary material, which is available to authorized users.

## Background

In the past decades, quality improvement has been considered by governments as a way to improve the effectiveness of Primary Health Care (PHC) systems, especially in Low and Middle Income Countries (LMICs), and extensive activities have been undertaken to improve the quality of these services [1]. Improving the effectiveness of services through providing high-quality services is one of the key factors for achieving Universal Health Coverage (UHC) [2]. The high quality of PHC services not only increases the effectiveness of cares but also the public’s trust on the health system [3–7]. Assessing the quality of service is the first step in quality improvement [8]. Quality assessment in PHC be able to use to improve performance through ensuring patient safety and health care providers responsibility for providing high-quality care, assessing and addressing gaps in how care is delivered and in health outcomes [9].

Some international organizations such as World Health Organisation (WHO) and the Organization for Economic Co-operation and Development (OECD) have been encouraging countries to measure and assess the performance and quality of their services, and they have presented some solutions in this regard [10–12]. Quality assessment in PHC may be much more complicated than other levels of the health system, as services are provided by a multi-expert team, and the performance of each individual in the team and the relationship between individuals and recipients of the services affect the final quality [13]. Using Quality Assessment Frameworks (QAF) is among the common methods for the assessment of PHC quality. The QAF serves as the foundation for quality improvement throughout the health organization. The QAF includes dimensions and Quality Indicators (QIs) related for each of them to be measured and monitored [14–17]. QIs are defined as a measurement tools of health care quality, which referring to the structures, processes and outcomes of care, can be used to monitor, assess and improve the quality of care, to compare service delivery units and to determine the quality of care trends [18, 19]. QAFs are usually developed and presented at national and international levels according to their conditions [14–17]. WHO Eastern Mediterranean Regional Office (EMRO) has announced seven dimensions of access, equity, safety, effectiveness, efficacy, patient centeredness and timeliness of services as quality dimensions in PHC. In this report, 34 QIs were introduced for assessing the quality [20]. The OECD has provided three key areas of health promotion, preventive care and diagnosis and treatment in primary care for assessing the quality of PHC [21]. The US Agency for Healthcare Research and Quality (AHRQ) has also proposed the dimensions of accessibility, coordination, efficiency, patient centeredness, effectiveness, safety, health system infra-structure and timeliness for quality assessment [22]. The existing differences in Primary Health Care Quality Assessment Frameworks (PHCQAFs) pinpoint the necessity for considering the needs, plans, goals and context of each country in the development of these QAFs. In addition, considering that the structure and services package of PHC in each country is different and they mainly depend on such factors as the community needs, economic power and the state of health system infrastructure [23, 24].

Every country should develop a national QAF to assess PHC in accordance with its own circumstances and characteristics. Regarding the PHC history in Iran and the brilliant results obtained from providing services in villages and cities which have been noted in WHO 2008 report [25], using QAF will strengthen primary health care system in Iran. Moreover, health transition and changing health needs in Iran elucidate the importance and necessity of QAF of PHC. This QAF can be implemented in first line (microsystem level) of service delivery to assessment of quality of services. The results of QAF implementation in first line can be used in policy/macro/meso level of system to set priorities, planning, policy-making by top-managers and policy-makers. In this regard, the aim of this study was to develop a QAF of PHC in Iran’s health system.

## Methods

**Fig. 1:**
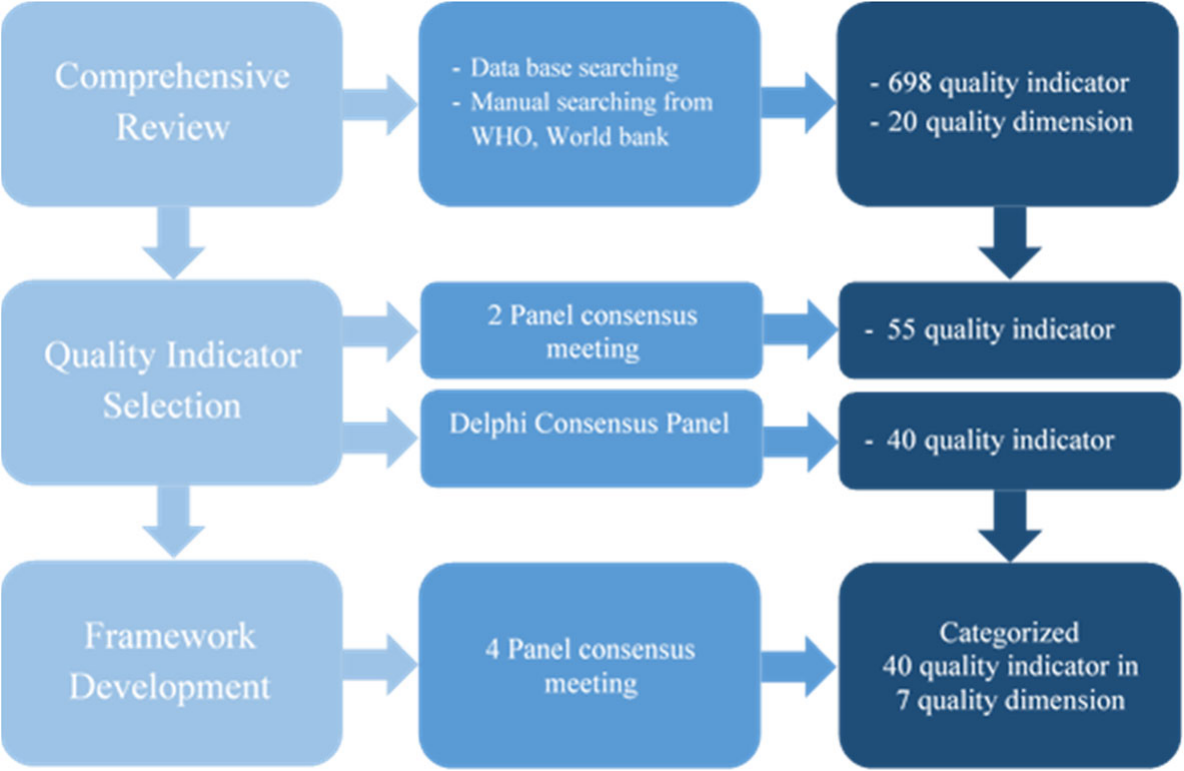
Iranian Primary Health Care Quality Assessment Framework Development flow

This study is a literature review which continued by a qualitative research in 2017. Three main phases including: literature review, selection of dimensions and QIs and developing Iranian PHCQAFs was performed (Fig. 1).

### Literature review

A comprehensive review of the PHCQAFs, dimensions and QIs was carried out through searching databases including Medline (PubMed), Science Direct, Scopus, Web of Science and Scientific Information Database (SID), Iran. The search was performed using various combinations and forms of the following search terms: ((primary health care[Title/Abstract] OR primary healthcare[Title/Abstract] OR primary care[Title/Abstract] OR primary health services[Title/Abstract] OR PHC[Title/Abstract])) AND (quality indicator*[Title/Abstract] OR quality index[Title/Abstract] OR quality dimension[Title/Abstract] OR quality domain[Title/Abstract] OR quality criterion[Title/Abstract] OR quality measure*[Title/Abstract] OR quality assess*[Title/Abstract] OR quality evaluat*[Title/Abstract]). Moreover, the websites of the WHO, World Bank, OECD, European Union and other related organizations were searched using the combination of these keywords. The search was limited with language (only Persian and English), time (from 1 January 2000 to 30 December2017) and full text availability.

The studies that introduced QIs in a QAF or as a set of quality dimensions were included in the study. Meanwhile, the studies that focused on a single indicator or a specific care quality (such as HIV screening) were excluded from the study. The retrieved studies were independently reviewed by two different researchers and the data were extracted from the selected articles based on a purposeful researcher-made form. In cases where there were disagreements between the two researchers, a third researcher intermediated. The extraction table contained the name of the author(s), the publication year, the country or organization providing the QAF, the quality dimensions and the QIs.

### Assessment and selection of QIs of Iran’s PHC system

A set of preliminary extracted QIs (698 QIs) was categorized by the research team using the information of the existing literature. In order to assess and select the final QIs, this collection was provided to the experts through holding panel meetings for the experts and also the Delphi method.

There were two entry criteria for the members of the panel sessions: holding a relevant academic degree (including health education, public health, epidemiology, management of health services, medicine and the like) and having at least 5 years of work experience in the field of PHC (working as chief executive in primary care at least at county level or other higher ranks such as vice chancellor for health, deputy vice chancellor for health and the like).

For the initial evaluation of QIs and quality dimensions, 2 panel sessions were held with the participation of 8 experts (1 health management specialist with 15 years of management experience in provincial and national levels, 3 general practitioners with 10 years of management experience in county and provincial levels, 1 psychiatrist with 15 years of management experience in provincial level, 2 specialists with 15 years of management experience in provincial level, 1 expert responsible for PHC monitoring and evaluation with 15 years of work experience). During these meetings, in addition to content analysis of the QIs, the relevance of the initial QIs to the local conditions of Iran, the coverage of current high-priority processes and their proportionality to the national PHC program were examined. Moreover, some QIs including special target group of age or sex were modified on Iran context. Finally, an initial list was extracted for countrywide assessment.

The countrywide assessment and prioritization of QIs was done through the Delphi method [26, 27]. The Delphi questionnaire/form was designed according to the comprehensive literature review and experts’ comments. At First step, the Delphi questionnaire, which included descriptions of the study objectives, the reasons for selecting participants, a form for collecting participant consent to complete the entire Delphi process, as well as the willingness to participate in the study and how to rate the QIs, along with the ability to make a comment and feedback for each QIs, was designed. In next step a Delphi questionnaires were sent by email for participants. The Delphi questionnaire was designed in such a way that the experts could assign an independent score ranging from 1 to 5 to each of the QIs in three dimensions of importance, relevance and feasibility in the healthcare system of Iran (Table 1) (see Additional file 1). The participants in the Delphi study included all health deputies of the country (35 participants) and the primary care experts of the Iranian Ministry of Health and Medical Education (MOHME) (4 participants).

**Table 1:** Scale rationing method for each quality indicator by 3 separate criteria

Preferred value	Importance	Relevance	Feasibility
1	Very little importance	Very low relevance	Very low feasibility
2	Little importance	Low relevance	Low feasibility
3	Medium importance	Medium relevance	Medium feasibility
4	Great importance	High relevance	High feasibility
5	Absolutely important	Absolutely relevant	Absolutely possible

After collecting the data, the average scores assigned to the QIs were calculated in terms of all the three dimensions on a scale of 100. Determining the priority of the QIs was carried out using the approach of the WHO EMRO office [20]. Therefore, the QIs with a final mean score of more than 70 were identified as the first priority; those with a mean score of 40 to 70 were identified as second priority; and the QIs with a mean score of less than 40 were excluded from the final QAF. Core indicators were ones which should be measured in all the provinces in meso levels and non-core indicators were ones which measured to provide additional information according to characteristics of the settings (see Additional file 2).

### Developing the QAF

To develop a QAF for Iran’s PHC system, 4 countrywide panel sessions were held at the MOHME with the participation of 35 specialists. Among these people, 5 experts from the MOHME, 3 PHC specialists at the MOHME, 20 health deputies of medical universities, the deputy vice chancellor for health of the MOHME and director of health network and 6 experts and professors in the field of health services management. In the panel sessions, the selected QIs of the Delphi study were evaluated according to 4 criteria as follows:

1. Relevance to national PHC programs
2. Global and national priorities in PHC
3. Maximum coverage of current PHC processes
4. The possibility of interventions to improve the QIs

Moreover, the quality dimensions extracted from the literature (20 dimensions) were discussed in the second session, and some dimensions were removed (5 dimensions) while some others were merged (8 dimensions). The classification of final QIs was done in the last panel session, based on approved dimensions.

## Results

### Literature review

Literature review led to the identification of 13 PHCQAF in the world. These QAF evaluated the quality of PHC in 20 dimensions and 698 QIs.

### Delphi survey and panel sessions

Reviewing the extracted QIs by the experts in terms of content and relevance to the local conditions of Iran led to a preliminary list of 55 QIs. Then, the selected QIs were evaluated through Delphi method by national level experts. Finally, 40 QIs (out of 55) were scored higher than 70 and preceded for review and finalization by the panel of experts.

The selected QIs were analyzed in terms of content and validity through four panel sessions, and some of them were reviewed and revised. The modifications were mostly related to the age group of the cares in different groups, and also the national service package in terms of how to provide the cares. The results of the review by the panel of experts led to the elimination of 2 QIs, revision of 11 QIs and adding of 2 other items to the QIs set. For determining the quality dimensions in the PHC system of Iran, the existing dimensions were examined, analyzed and then matched with the selected QIs in another panel session. Resultantly, 20 dimensions extracted from the literature were briefed and finalized in 7 dimensions.

So, the dimensions of health promotion, economic conditions/expenditures, diagnosis and treatment: primary care, health status and health system infra-structure/information technology were eliminated due to non-matching with Iran’s primary care system. Moreover, the dimension of continuity was merged with the dimension of comprehensiveness, preventive care with effectiveness and workforce development with safety. In addition, the dimension of accessibility and equity in health was categorized under the joint dimension of “access and equity”. Also, the dimensions of comprehensiveness, timeliness, acceptability and coordination in the global QAFs were identified as appropriateness dimension within the QAF of Iran. Finally, 40 QIs for assessing the quality of PHC were categorized by the panel of experts in the form of 7 dimensions of quality. These dimensions were: access and equity, safety, efficacy, effectiveness, patient-centeredness, governance, and appropriateness) (Table 2).

**Table 2:** Quality Evaluation QIs in Iran’s Primary Health Care

Domains	Level	Indicators	Relevance	Importance	Feasibility	Total	references
Access and equity	Structure	1. % of catchment population who received at least one basic visit	84.71	87.06	80.59	84.12	[28]
	Process	2. % of patients with mental disorders that have had a follow-up visit in defined period according to national protocol.	80.54	82.16	68.65	77.12	[29]
	Outcome	3. % of pregnant women with first visit at the first trimester	94.74	92.63	91.58	92.98	[20]
		4. % of population, age 30 to 59 years old with overweight and obesity who received consultation services for behavioral change	90.00	89.47	69.47	82.98	[22]
		5. % of smokers, age 18 and older who receive smoking cessation consultation	89.71	88.57	62.29	80.19	[30, 31]
		6. % of student age 6 to 14 years old who received florid therapy	90.27	89.19	88.65	89.37	expert panel
		7. Clinical staff provide home visits for patients who are physically not able to travel to the practice	76.84	77.37	56.84	70.35	[32]
		8. % of individuals with COPD that have had a follow-up visit and treatment during the last year.	79.46	79.46	63.24	74.05	[14]
		9. Individual self-care program coverage	Final Expert Panel				
safety	Process	10. % of health facility staff immunized for Hepatitis B (3 doses).	84.74	77.89	89.47	84.04	[33]
		11. % of safe injections in the health care facility	81.05	82.63	73.16	78.95	[34, 35]
		12. % of Staff who have attended continuous training about quality and patient safety during last year	77.78	78.89	79.44	78.70	[36, 37]
		13. % compliance with Hand Hygiene guidelines	81.03	77.95	73.33	77.44	[38, 39]
	Outcome	14. Number of adverse events reported (immunization/medication)	87.18	88.21	80.51	85.30	[40, 41]
Efficiency	Process	15. % of prescriptions that include antibiotics in health centers and health posts	77.89	78.42	69.47	75.26	[42]
	Outcome	16. % of the 13 essential non communicable diseases medicines with no stock out in last 3 months (cardiovascular, diabetes, hypertension and COPD)	82.29	80.57	73.14	78.67	[43]
Effectiveness	Outcome	17. % Hypertension patients with Initial laboratory investigations	80.00	81.05	67.37	76.14	[44–46]
		18. % of registered hypertension patients with BP < 140/90 at last 2 follow up visits	91.28	90.26	78.46	86.67	[47]
		19. % of registered diabetic patients with fasting blood sugar controlled at last 2 follow up visits	94.36	94.36	81.03	89.91	[48, 49]
		20. % of registered NCD patients age 30 and older with 10 years’ cardiovascular risk recorded in past 1 year	95.90	92.82	78.97	89.23	[43, 50]
		21. % of registered NCD patients with blood pressure recorded twice at last follow up visit	93.33	93.33	83.08	89.91	[43, 51]
		22. % children age 6 to 9 months old screened for anemia	85.64	86.67	71.28	81.20	[52, 53]
		23. % of women who delivered and received at least once postnatal care within the first 6 weeks	92.31	90.77	82.05	88.38	[54]
		24. % of under 23 months children immunized according to the national protocol	96.92	97.44	95.90	96.75	[55]
		25. % of people that work in the workshop of under 20 labor worker and whose were basic visit and occupational care in past years.	Final expert panel				
		26. % of diabetic people with HbA1C less than 7%	92.82	90.77	75.90	86.50	[56, 57]
		27. % of pregnant women received at least 6 ANC	89.73	86.49	80.54	85.59	[58]
		28. % of under 5 children that had weight and height measured in past 1 year	96.41	93.33	92.82	94.19	[59]
		29. % of newborns who are exclusively breastfed for the first six months	93.85	93.33	85.64	90.94	[54]
Patient centeredness	Outcome	30. % of patients aware about Patients’ rights and responsibilities	77.30	75.68	58.92	70.63	[60, 61]
		31. Customer satisfaction rate (%)	82.22	87.22	63.89	77.78	[62]
governance	Outcome[55]	32. Staff satisfaction rate	84.24	86.06	69.09	79.80	[63]
		33. % of appropriate (upward) referrals during last 6 months (by specific conditions) with appropriate feedback	82.70	82.16	62.16	75.68	[64, 65]
appropriateness	Process	34. % of population, age 30 and older, with diabetes mellitus who received following exams: • Hemoglobin A1c (Hba1c); • Eye examination; • Foot Examination; • Blood Pressure Measurement	92.97	93.51	71.89	86.13	[66]
		35. % of population, age 20 and older, with depression who received following exams: • PHQ • Active follow up • non-communicable diseases risk factors assessment • Drug Side effects assessment	83.68	87.37	62.11	77.72	expert panel
		36. % of pregnant women who received health education about: • nutritional care • anemia • sanitation • high risk pregnancy signs	94.87	93.85	84.10	90.94	[67–69]
	Outcome	37. % TB screening in high risk groups	95.14	92.43	83.78	90.45	expert panel
		38. % of women aged 30–59 yrs. who had at least 1 Pap test in the past 5 yrs.	87.37	86.32	82.11	85.26	[14]
		39. % of risk factors Assessment for AIDS in The population covered.	92.97	93.51	70.27	85.59	expert panel
		40. % of water microbial sampling According to standard.	93.51	90.81	90.27	91.53	expert panel

**Fig. 2:**
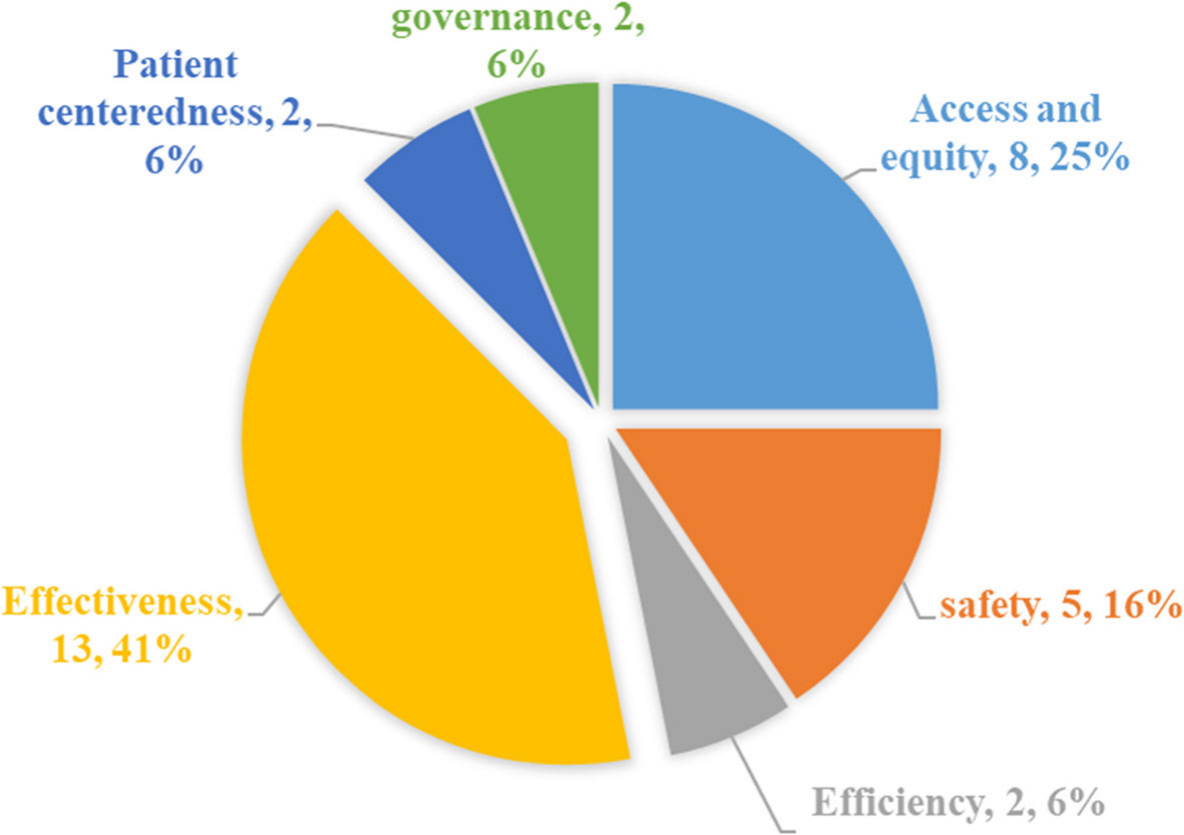
The shares of each dimension in the number of quality indicators

Out of 40 QIs for quality assessment in Iran’s PHC system, 33.5% (13 QIs) were related to the dimension of effectiveness. This dimension had the highest share among the quality dimensions. Meanwhile, each of the dimensions of patient-centeredness, efficiency and governance had only 5% (2 QIs) of share. So, they had the lowest shares (Fig. 2).

## Discussion

In this study, through using a number of valid and scientific methods (literature reviews, Delphi method and panel of experts), the national PHCQAF in Iran was developed. The QAF was eventually finalized with 40 QIs in the form of 7 dimensions of quality, including access and equity, safety, efficacy, effectiveness, Governance, patient*-*centeredness and appropriateness.

The OECD has developed 26 QIs in 3 general dimensions of health promotion, preventive care, diagnosis and treatment: primary care and health status, which is completely different with Iran’s QAF in terms of quality dimensions. The international approaches of this organization as well as the type of primary care delivery system in the member states justify this difference. However, the national QAF of Iran has many similarities with the QAF provided by EMRO and QAF of such countries as Australia, Canada and the United States in terms of the classification of dimensions and QIs. All these QAF have 3 dimensions in common: safety, access and effectiveness. Though, there are some differences in the classification of QIs in terms of dimensions between the QAF of Iran and the mentioned QAFs [20–22, 66, 70–72]. The classification of QIs in terms of dimensions in the QAF of Iran was carried out by the experts according to the content of each QI. Given the existing similarity between the QIs presented under two separate dimensions of access and equity in different QAFs, the experts merged these two dimensions and developed the Iranian QAF with a single dimension entitled access and equity, which is similar to the QAF developed by EMRO [20].

The developed dimensions of Iran’s QAF provide a comprehensive coverage for the quality in primary health care. The QIs in this QAF have been developed based on the structure of service providing in PHC, available services packages and country conditions. In this way, most of the PHC plans in Iran are covered and it is concentrated on health promotion, prevention and outpatient treatment level of services.

According to the Donabedian quality model (Donabedian A 1988) which has been represented by 3 categories: structure, process and outcome, the majority of the developed QIs (72.5%) are related to the outcome category which mostly focused on effectiveness, people centeredness and governance. This could lead to more focus on public-centeredness in service supplying, effectiveness of the services and effective coverage of them in the PHC system which are highly emphasized by the WHO; these are also some determining factors in the evaluation of the functions of health care systems [73, 74]. Measurement and tracking of these QIs in PHC system could also lead to increases in the utilization of evidence-based protocols and guidelines in providing primary health care services, and the effectiveness of these services in responding to people needs.

Within Iran’s PHCQAF, seven unique QIs have been developed exclusively based on the current service package in primary health care system and the burden of common diseases in Iran which is not observed in other similar QAF. Six of these QIs (which include oral hygiene, self-care plan, AIDS screening, mental health, TB screening and quality of drinking water) were related to accessibility and appropriateness dimensions of PHC quality. These two dimensions are taken into consideration by participating experts in this study, due to their undeniable impact on the coverage and concentration on care process and continuous improvements of its quality, since the process improvements will eventually bring out valuable outcomes for the public.

Self-care plan coverage is one of the unique QIs in Iranian PHCQAF. Considering the diseases’ trending in Iran reveals the growing increase of chronic diseases which mostly are due to unhealthy lifestyle of people with their increasing tendency toward unhealthy eating, inactivity and smoking [75, 76]. Self-care plan are one of the most important primary health care programs regarding its role in promoting public health literacy and skills to employ healthy behaviors, which are fundamental ways of preventing chronic diseases.

Reviewing health and hygiene problems in Iran shows that oral hygiene is a serious case especially among children and teenagers [77]. Dental services including fluoride therapy and more specialized services like fissure sealants have been merged in the primary health care service packages after evolutions of health care reform plans in Iran [78]. Regarding the non-coverage of preventive and treatment dental services by basic health insurances, and the importance of them for the health of individuals and society, choosing the QI of fluoride therapy among students coverage could lead to improvement of consistency and quality of this plan which will eventually upgrade the DMFT QIs among children and teenagers.

Mental health and the risk of cardiovascular diseases Screening QI is another special QI in Iran’s QAF. Nowadays, cardiovascular diseases are the cause of almost half (46%) of the death rates among Iranians (2014), and they are the cause of 16% YLL in Iran (2010, [79–81]). In addition, the spread of mental illnesses and a lack of sufficient attention to them in Iran’s primary health care system have led to more focus on this field in the recent health care reform plan. The new mental health plan has been merged into Iran’s primary health care services package [78]. Screening, providing active service and continuous patient tracking is on the agenda in the new defined services. It seems that merging QIs related to mental health care and tracking them will cause more attention and concentration on mental health services quality improvement.

The QAF could be used as a comprehensive tool in Iran’s primary health care system for national, regional and local levels of constant tracking and improvements of service quality. This QAF could also be used as a tool to compare different functioning layers of the primary health care system. The evaluation of PHC services quality through QAF in different levels is an opportunity to identify the weaknesses and challenges. It is obvious that this would create necessary base in which evidence-based planning is taken for granted in order to alter resource allocation process, reengineering the processes, defining new standards and using evidence-based guidelines. Furthermore, developing and using QAF in primary health care systems of different countries, especially low and middle income countries, will provide settings for comparison and modeling of the actions and plans of successful countries.

Considering that Iran’s PHC system is undergoing structural and performance reforms, the national QAF for primary health care can provide an efficient tool for policy makers to manage the plans. This QAF defines the scope and dimensions of performance evaluation. Meanwhile, it makes balance between the performance evaluation system, policy priorities and funding institutions, and provides a clear and shared vision of performance for both the service providers and customers. Also, given that this QAF has been developed with the participation of national policy makers of Iran’s PHC system -as the final users of the QAF data- its credibility and application will be in place for the future policy making programs.

Lack of patients’ involvement in the process is one of the study limitations. Also, as the indicators were developed and selected in meso level, we expect this limitation to have not a considerable effect on the results.

## Conclusion

The present study was designed in accordance with the relevant global and regional evidences and with the participation of PHC experts and managers at national and regional levels in Iran. It was developed for the current situation of the Islamic Republic of Iran and is fully in line with the new PHC structure and the services packages developed for the healthcare evolution plan. The developed QAF, as one of the main steps in continuous quality improvement, can play a vital role in continuous assessment and improvement of the quality of PHC system. Therefore, it is recommended to use this QAF as a tool for qualitative assessment of the Iranian PHC system performance, including the service providers at national and provincial levels, and move towards the establishment of a continuing program for quality improvement of the services and also provision of universal health coverage. Resultantly, this will promote the health status of the community and make the service providers satisfied. Using this QAF in the developing countries, and especially in Iran, results in strengthening the health system and its accountability. Also, the QAF developed in this study can serve as a model for other developing countries in designing their own national QAFs.

## Additional files

Additional file 1: Delphi questionnaire/ form example (DOCX 26 kb)

Additional file 2: List of None-Core Quality indicators (DOCX 34 kb)

## Data Availability

All data generated or analyzed during this study are included in this published article.

## Abbreviations

EMRO: Eastern Mediterranean Regional Office
LMICs: Low and Middle Income Countries
OECD: Organization for economic co-operation and development
PHC: Primary health care
PHCQAF: Primary health care quality assessment frameworks
QAFs: Quality Assessment Frameworks
QIs: Quality Indicators
UHC: Universal Health Coverage
WHO: World Health Organization
