# Supplementary material for: Developing Iranian primary health care quality framework: a national study
Source: BMC Public Health. 2019 Jul 9;19:911. doi: 10.1186/s12889-019-7237-8 (PMC6617563; doi:10.1186/s12889-019-7237-8)
Supplement: Supplementary file 1 — Delphi questionnaire/ form example (DOCX 26 kb) [file 12889_2019_7237_MOESM1_ESM.docx]

Additional file 1:

Appendix1- Delphi questionnaire/ form example

| **Dimension: Access and Equity** | | | | | | | | | | | | | | | | |
| --- | --- | --- | --- | --- | --- | --- | --- | --- | --- | --- | --- | --- | --- | --- | --- | --- |
| Code=1-1-1 | | | | | Title= Percentage of catchment population who received at least one basic visit | | | | | | | | | | | |
| Guide | | | | | Knowledge of healthcare facility’s catchment area is important for assessing health service utilization, for calculating population-based rates of disease and for performing other important analysis. | | | | | | | | | | | |
| **Calculating=** Number of individual patient visits for the PHC facility coming from a specific area over a set time period / Population size in this area | | | | | | | | | | | | | | | | |
| **Your comments:** | | | | | | | | | | | | | | | | |
| **Importance** | | | | | | **Relevance** | | | | | **Feasibility** | | | | | |
| **1** | **2** | **3** | **4** | **5** | | **1** | **2** | **3** | **4** | **5** | | **1** | **2** | **3** | **4** | **5** |
|  |  |  |  |  | |  |  |  |  |  | |  |  |  |  |  |
