# Supplementary material for: Developing Iranian primary health care quality framework: a national study
Source: BMC Public Health. 2019 Jul 9;19:911. doi: 10.1186/s12889-019-7237-8 (PMC6617563; doi:10.1186/s12889-019-7237-8)
Supplement: Supplementary file 2 — List of None-Core Quality indicators (DOCX 34 kb) [file 12889_2019_7237_MOESM2_ESM.docx]

Additional file 2: List of None-Core Quality indicators

| **Indicators** | Relevance | Importance | Feasibility | Total |
| --- | --- | --- | --- | --- |
| 1. Number of days of stock outs per year for identified 15 essential medicines in the available EDL in the facility | 68.58 | 68.49 | 68.33 | 68.47 |
| 1. % of substance users including tobacco users in receipt of brief intervention | 75.59 | 73.59 | 58.42 | 69.20 |
| 1. % of high risk group immunized against Influenza | 71.26 | 74.21 | 64.21 | 69.89 |
| 1. Average waiting time (min) at out-patient clinics | 79.78 | 76.11 | 62.22 | 69.37 |
| 1. Average length of pc provider patient relationship | 70.23 | 73.33 | 64.67 | 69.41 |
| 1. Appropriate sputum’s specimens | 64.53 | 61.58 | 76.67 | 67.59 |
| 1. Epidemiological data set: Are clinical patient records from GP/PC used at regional or local level to identify health needs or priorities for health policy? | 72.56 | 78.38 | 58.92 | 69.95 |
| 1. Depression screening for pregnant and post–partum   women | 65.23 | 67.58 | 68 | 66.94 |
| 1. Prescription for antidepressant in patients with depression in past year | 76.32 | 60.23 | 61.58 | 66.04 |
| 1. The Organizational Trust rate | 65.69 | 76.36 | 59.39 | 67.15 |
| 1. percentage of decisions that made by the Board of Trustees health centers | 74.84 | 65.89 | 59.35 | 66.69 |
| 1. health data quality | 68.45 | 63.12 | 72.12 | 67.90 |
